# Sputtering Coating of Lithium Fluoride Film on Lithium Cobalt Oxide Electrodes for Reducing the Polarization of Lithium-Ion Batteries

**DOI:** 10.3390/nano11123393

**Published:** 2021-12-14

**Authors:** Shasha Qu, Wenbin Wu, Yunfan Wu, Yanping Zhuang, Jie Lin, Laisen Wang, Qiulong Wei, Qingshui Xie, Dong-Liang Peng

**Affiliations:** College of Materials, State Key Laboratory for Physical Chemistry of Solid Surfaces, Fujian Key Laboratory of Materials Genome, and Collaborative Innovation Center of Chemistry for Energy Materials, Xiamen University, Xiamen 361005, China; qshaashaa@163.com (S.Q.); webb523@163.com (W.W.); wyf-0825@163.com (Y.W.); zhuangyanpingya@163.com (Y.Z.); qlwei@xmu.edu.cn (Q.W.); dlpeng@xmu.edu.cn (D.-L.P.)

**Keywords:** voltage polarization, lithium cobalt oxide, lithium fluoride, lithium-ion battery

## Abstract

Lithium cobalt oxide (LCO) is the most widely used cathode materials in electronic devices due to the high working potential and dense tap density, but the performance is limited by the unstable interfaces at high potential. Herein, LiF thin film is sputtered on the surface of LCO electrodes for enhancing the electrochemical performance and reducing the voltage polarization. The polarization components are discussed and quantified by analyzing the relationship between electrochemical polarization and charger transfer resistance, as well as that between concentration polarization and Li-ion diffusion coefficients. In addition, the decreased charge transfer resistance, increased lithium-ion diffusion coefficients, and stabilized crystal structure of LiF-coated LCO are confirmed by various electrochemical tests and in-situ XRD experiments. Compared to that of pristine LCO, the capacity and cycling performance of LiF-coated LCO is improved, and the overpotential is reduced upon cycling. This work provides reference for quantifying the various polarization components, and the strategy of coating LiF film could be applied in developing other analogous cathode materials.

## 1. Introduction

Lithium-ion batteries (LIBs) are widely used in energy storage equipment due to their high energy density, good cycling stability, and environmental friendliness [1]. The main limitation for the overall capacity of LIBs is the low capacity of cathode materials including LiCoO_2_ (LCO) [2,3], LiFePO_4_ [4], LiMn_2_O_4_ [5], and Ni-rich oxides [6]. Although the cathodes have been commercialized over the years, LCO still occupies the dominant position in portable electronics due to the high theoretical capacity, stable cycling performance, high working potential, and facile synthesis [7,8]. LCO cathode has a theoretical capacity of ~140 mAh g^−1^, in which 0.5 Li^+^ could be extracted per LCO reversibly when charged to 4.2 V [9]. Charging to higher cut-off voltage could extract more Li^+^ reversibly to increase the reversible capacity of LCO [10]. Unfortunately, the dramatic structural collapse [11] and severe side reactions with liquid electrolyte [12] always result in the fast capacity and voltage attenuation.

Upon cycling, the side reactions in the interfaces between cathode and electrolyte aggravate and the structure of LCO gradually deteriorate. Protecting the surface of LCO electrodes is one of the most effective methods to mitigate the interfacial reactions. Since LiPF_6_ reacts with H_2_O to produce LiF, POF_3_, and HF, the coatings including metal oxides (MgO [13], ZnO [14], ZrO_2_ [15], TiO_2_ [16], etc.) and fluorides (AlF_3_ [17], LiAlF_4_ [18], LiF [19], etc.) can enhance the interfacial stability of LCO in the LiPF_6_-based liquid electrolyte. Lee et al. [3] confirmed that AlF_3_ or LaF_3_ coating on the surface of LCO can inhibit the corrosion by the liquid electrolyte directly. Myung et al. [20] proposed that metal-fluoride layer can avoid the corrosion of the LCO electrode by the HF gas generated from the decomposition of liquid electrolyte. With wide electrochemical stability window of ~6.3 V, high interfacial energy to Li (73.28 meV Å^−2^), and low electronic conductivity [21], LiF is expected to be an ideal surface coating material for cathode materials. As a side production of liquid electrolyte, LiF can also buffer the further decomposition of liquid electrolyte. Using atomic layer deposition [22], plasma laser deposition [23], magnetron sputtering [14], and other physical vapor deposition routes, the uniform and pure thin films can be coated on the electrode surface. Among them, magnetron sputtering has distinct characteristics of stable deposition speed and uniform morphology.

Despite the tremendous efforts to increase the specific capacity and durability of LCO cathodes [24], few reports study about increasing the energy efficiency (charge/discharge energy) or decreasing the voltage hysteresis (charge/discharge voltage) by coatings. As a result, herein radio-frequency (RF) magnetron sputtering is utilized to fabricate the LiF-coated LCO electrodes for LIBs. Specifically, the LiF coating can reduce the concentration polarization and electrochemical polarization of LCO electrodes, which are confirmed by theoretical analysis and experimental tests. Compared to that of the pristine LCO, the LiF-coated LCO exhibits higher capacity, better cycling performance, and lower overpotential upon cycling. The results are ascribed to the increased Li-ion diffusion coefficients, decreased charger transfer resistance, and suppressed interfacial reaction by the LiF coating.

## 2. Experimental

### 2.1. Sample Fabrication

**Figure 1:**
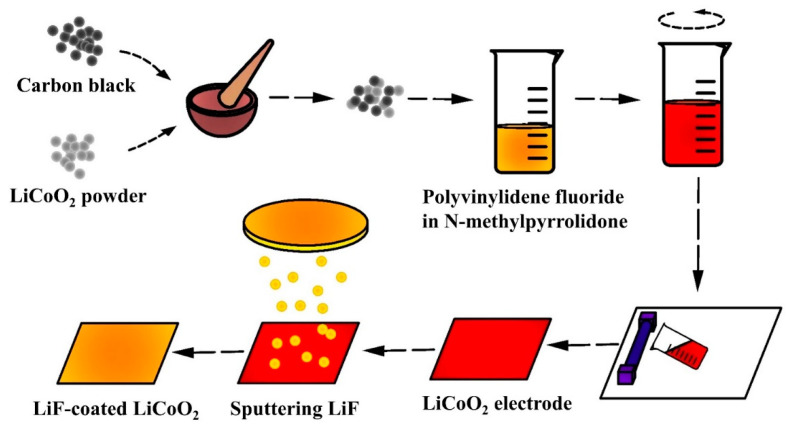
Fabrication process of LiF-coated LCO electrodes by casting and sputtering.

As schematically illustrated in Figure 1, commercial LiCoO_2_ powder (Aladdin, China) was mixed with conductive carbon black and polyvinylidene fluoride (PVDF) binder at a mass ratio of 8:1:1 and dissolved in N-methylpyrrolidone (NMP) solvent (Duoduo, China). The slurry was coated on Al foils and dried in vacuum oven at 120 °C for 12 h to obtain the pristine LCO sample. LiF film was coated on the pristine LCO electrode by sputtering a 6-inch LiF target to fabricate the LiF-coated LCO sample. The pure Ar gas was pumped into the chamber as the working gas. The LiF films were deposited at the working pressure of 0.5 Pa for 40 min. The substrate was rotated at a speed of 10 r min^−1^ to ensure the uniform coating.

### 2.2. Materials Characterization

The surface morphology characteristics of LCO electrodes with or without LiF coatings were observed by field emission scanning electron microscope (FESEM, HitachiSU-70, Japan Hitachi High-Tech Naka Business, Tokyo, Japan). The X-ray energy dispersive spectrum (EDS) was used to analyze the distribution and content of elements in the sample microregion. The X-ray diffraction instrument (Bruker-Axe X-ray diffractometer, Karlsruhe, Germany, Cu Kα radiation, 40 kV, 40 mA) was used to analyze the crystal structure and phase composition of LCO films. The self-made battery was charged and discharged using a battery system with a current density of 0.2C during the in-situ XRD experiment, and the XRD patterns were collected for every 10 min. Surface chemical compositions analysis were measured by X-ray photoelectron spectroscopy (XPS) using Thermo Scientific K-Alpha spectrometer (Thermo Fisher Scientific, Waltham, MA, USA) and monochromatic Al-Kα (*hv* = 1486.7 eV). High-resolution spectra are obtained with 20 eV constant pass energy, and the analyzed area was about 300 μm × 700 μm. All spectral energies were standardized with reference to the C1s peak at 284.8 eV.

### 2.3. Electrochemical Tests

The electrochemical properties of LCO and LiF-coated LCO electrodes were tested by CR2025-type coin cells which were assembled in Ar-filled glovebox. Li foil was used as the counter electrode, and polypropylene membrane was used as the separator. The 1 M LiPF_6_ dissolved in a mixture of ethylene carbonate/ethyl methyl carbonate/dimethyl carbonate (EC: EMC: DMC = 1:1:1 in volume) was used as the liquid electrolyte. The assembled cells were aged at room temperature for 24 h. The galvanostatic charge and discharge tests were conducted on the Neware batteries testing system (BT2000). The voltage range of charge/discharge was 2.8–4.2 V at 0.2 C (firstly activating 3 cycles at 0.05 C). The electrochemical impedance spectra (EIS) were tested on a Princeton electrochemical working station (PARSTAT 3000A) with the frequency range of 0.01–1 MHz and with an amplitude voltage of 5 mV. The CV tests were performed from 2.8 to 4.2 V at a sweep rate of 0.1 mV s^−1^ using an electrochemical working station (CHI660e). Galvanostatic intermittent titration technique (GITT) measurements were tested from 2.8 to 4.2 V, in which 0.1 C rate current was applied for 10 min and then relaxion was set for 4 h.

## 3. Results and Discussion

**Figure 2:**
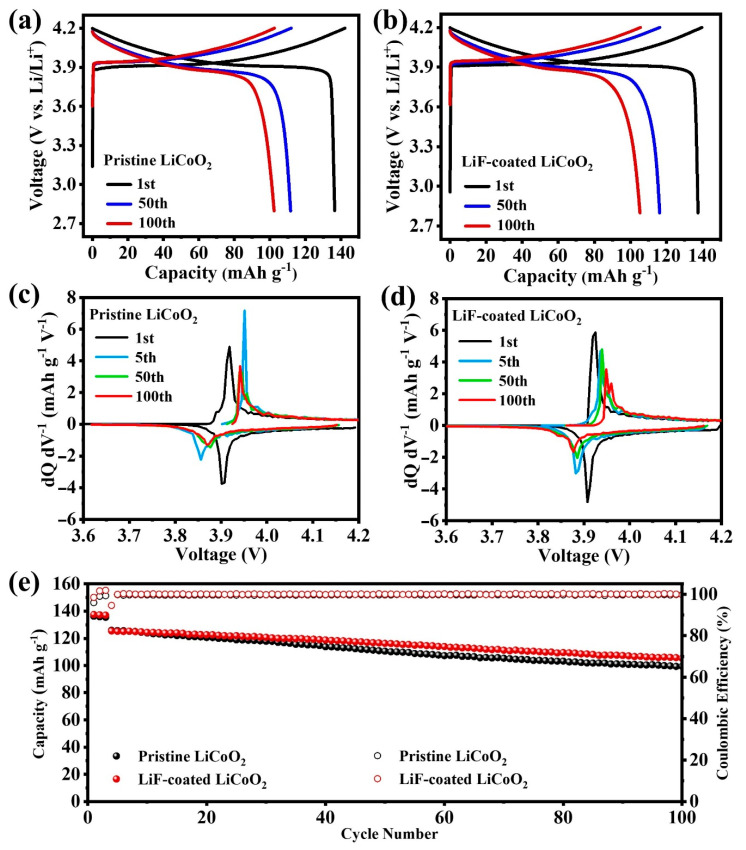
(**a**,**b**) Charge/Discharge curves, (**c**,**d**) differential capacity plots, (**e**) cycling performance and corresponding Coulombic efficiencies of pristine LCO and LiF-coated LCO.

Figure 2a,b show the voltage profiles of pristine LCO and LiF-coated LCO in the 1st, 50th, and 100th cycles. The two electrodes exhibit comparable capacity and similar voltage profiles in the first three cycles, but LiF-coated LCO exhibits higher initial Coulombic efficiency (CE) (98.48%) than that of pristine LCO (95.92%). Afterwards, the voltage polarization of LiF-coated LCO is lower than that of pristine LCO. In the 50th cycle, the discharge capacity of LiF-coated LCO and pristine LCO is 116.2 and 110.5 mAh g^−1^, respectively. After 100 cycles, the discharge capacity of LiF-coated LCO (105.8 mAh g^−1^) keeps higher than that of pristine LCO (99.3 mAh g^−1^). Figure 2c,d present the capacity differential plots of pristine LCO and LiF-coated LCO with similar current density and peak location in the first cycle. In the 1st, 5th, 50th, and 100th cycles, the overpotential of pristine LCO is 0.010, 0.089, 0.066, and 0.075 V, while that of LiF-coated LCO is 0.010, 0.049, 0.051, and 0.070 V, demonstrating the reduced volage polarization of LiF-coated LCO upon cycling.

The reversible capacity and CEs of LiF-coated LCO remain higher than those of pristine LCO all along the cycling process (Figure 2e), and the capacity retention of LiF-coated LCO (76.9%) is also higher than pristine LCO (73.6%) after 100 cycles. The improvement is ascribed to the protective effects of LiF coating, which enhances the Li-ion diffusion kinetics and decreases the charge transfer resistance, leading to the reduced voltage polarization. The improved Li-ion diffusion and charger transfer kinetics will be further confirmed by the tests below. The improved capacity and cycling stability could be advantageous for regulating the practical application of cathode materials in LIBs.

**Figure 3:**
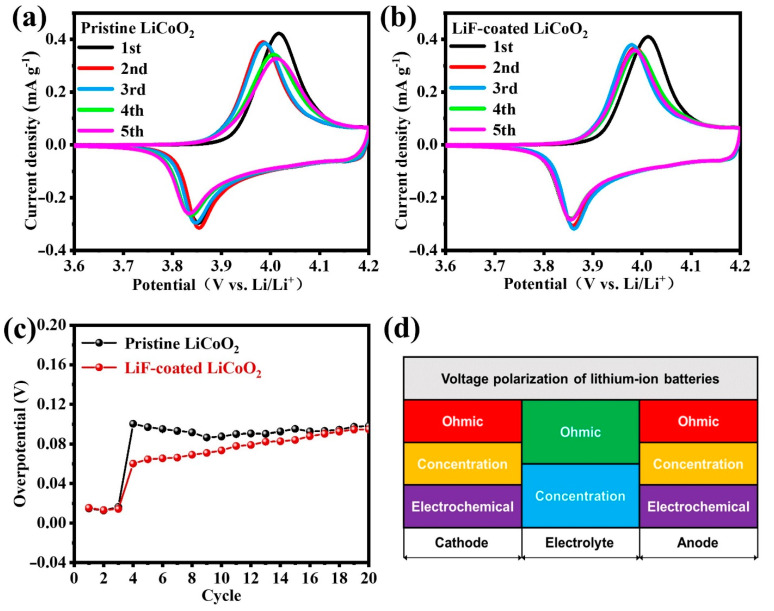
(**a**,**b**) Cyclic voltammetry curves of pristine LCO and LiF-coated LCO. (**c**) Overpotential of average voltage of pristine LCO and LiF-coated LCO. (**d**) Voltage polarization of lithium-ion batteries for cathode, electrolyte, and anode components.

CV curves are evaluated to compare the voltage polarization of the two electrodes as shown in Figure 3a,b. The (de)intercalation potential is mainly located between 3.6 and 4.2 V. No obvious differences can be found in the peak potential of redox reactions between pristine LCO and LCO-LiF electrodes in the first cycle which are corresponding to the phase transition between the two O3 phases of hexagonal and monoclinic [25]. However, the oxidation peaks of pristine LCO keep shifting, but the peak position of LiF-coated LCO remains stable. In addition, the width of oxidation peaks of pristine LCO are wider than that of LiF-coated LCO, which is indicative of the unstable crystal structure upon cycling. Figure 3c shows the overpotential of average voltage which is calculated from Figure 2e. The overpotential of pristine LCO is 0.10 V in the 4th cycle, while that of LiF-coated LCO is only 0.06 V. Moreover, it is apparent that the overpotential of pristine LCO remains higher than that of LiF-coated LCO in the first 20 cycles, indicating the stably reduced voltage polarization by the surface coating of LiF. Considering the various polarization components as shown in Figure 3d, the concentration and electrochemical polarization of the two samples are investigated by comparing the charge transfer resistance and Li-ion diffusion coefficients as to be discussed below.

The electrochemical overpotential can be calculated based on Equation (1): [26]

(1) $$\eta_{e}=-\frac{\mathrm{RT}}{\alpha F}lnj^{0}+\frac{\mathrm{RT}}{\alpha F}ln\frac{I}{A}$$

where R is the gas constant, T is the absolute temperature, *α* is the transfer coefficient, F is the Faraday constant, *j*^0^ is the exchange current density, *I* is the current, and *A* is the surface area of electrode. By derivatizing the electrochemical overpotential (*η*_e_) with the current (*I*), the electrochemical resistance can be obtained as Equation (2):

(2) $$\frac{d\eta_{e}}{dI}=\frac{\mathrm{RT}}{\alpha FI}$$

The charge transfer resistance can be expressed as Equation (3):

(3) $$R_{\mathrm{ct}}=\frac{\mathrm{RT}}{Fj}$$

where *j* is the current density and equals to *I*/*A*. Consequently, the electrochemical polarization of different electrodes can be quantified by comparing the charger transfer resistance (*R*_ct_) tested from electrochemical impedance spectrum (EIS).

Similarly, the concentration overpotential can be calculated based on Equation (4):

(4) $$\eta_{c}=-\frac{\mathrm{RT}}{nF}\ln\left(1-\frac{I}{Aj_{d}}\right)$$

where *n* is the charge transfer number, and *j*_d_ is the limiting current density. By derivatizing the concentration overpotential (*η*_c_) with the current (*I*), the concentration resistance can be obtained as Equation (5):

(5) $$\frac{d\eta_{c}}{dI}=\frac{\mathrm{RT}}{nF\left(Aj_{d}-I\right)}$$

The limiting current density (*j*_d_) is calculated based on Equation (6):

(6) $$j_{d}=nFD\frac{c^{0}}{l}$$

where *D* is the Li-ion diffusion coefficient, *c*^0^ is the ionic concentration on the electrode surface, and *l* is the diffusion length. Consequently, the concentration polarization of different electrodes can be quantified by comparing the Li-ion diffusion coefficient (*D*) tested from Galvanostatic intermittent titration technique (GITT).

**Figure 4:**
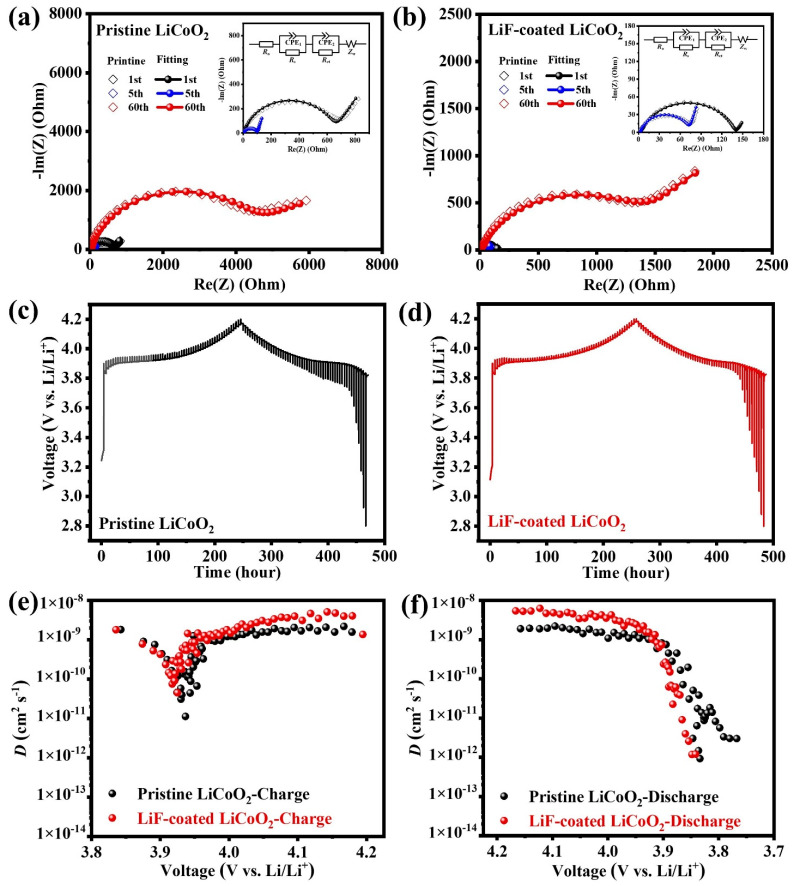
Electrochemical impedance spectrum of (**a**) pristine LCO and (**b**) LiF-coated LCO after the 1st, 5th, and 60th cycles. (**c**,**d**) Galvanostatic intermittent titration curves and (**e**,**f**) corresponding Li-ion diffusion coefficients of pristine LCO and LiF-coated LCO.

The EIS plots are tested to compare the electrochemical polarization of pristine LCO and LiF-coated LCO upon cycling as shown in Figure 4a,b. The EIS plots are composed of a semicircle and a diagonal line. As depicted in the insets of Figure 4a,b, the semicircle at high frequency is related to the charge transfer at the cathode and electrolyte interface, and the diagonal line at low frequency is determined by the diffusion of Li-ions in the electrode called Warburg impedance. Based on the fitting results, the charge transfer resistances of pristine LCO (693, 104, 4483 Ω) are all higher than those of LiF-coated LCO (140, 76, 1340 Ω) in the 1st, 5th, and 60th cycles. Miyashire [27] proposes that the capacity attenuation of LCO during cycling is mainly due to the increase in the interface resistance between cathode and electrolyte, which is related to the existence of the passivation layer. As a result, the decreased interfacial resistance of LiF-coated LCO implies that the LiF coating effectively reduces the electrochemical polarization upon cycling.

The GITT tests are used to compare the concentration polarization of pristine LCO and LiF-coated LCO in the initial cycle as shown in Figure 4c,d. The two electrodes both exhibit the similar voltage plateaus but the fluctuation voltage of LCO is obviously large than that of LiF-coated LCO when the charging voltage is higher than 3.9 V. Meanwhile, the polarization degree of pristine LCO increases drastically upon discharge, while that of LiF-coated LCO remains stable when the discharge voltage is above 3.9 V. The Li-ion diffusion coefficient (*D*) measured by GITT is calculated based on Equation (7): [28]

(7) $$D=\frac{4L^{2}}{\pi \tau }{\left(\frac{\Delta E_{s}}{\Delta E_{t}}\right)}^{2}$$

where τ is the relaxation time, *L* is the Li-ion diffusion length and equals to the thickness of electrode, Δ*E*_s_ is the steady-state voltage change by the current pulse, and Δ*E*_t_ is the voltage change during the constant current pulse after eliminating the *iR* drop. Figure 4e,f show the Li-ion diffusion coefficients of the two electrodes which are derived from Figure 4c,d. The Li-ion diffusion coefficients of LiF-coated LCO are all higher than that of pristine LCO above 3.9 V, implying the reduced concentration polarization of LiF-coated LCO upon the redox reaction process.

**Figure 5:**
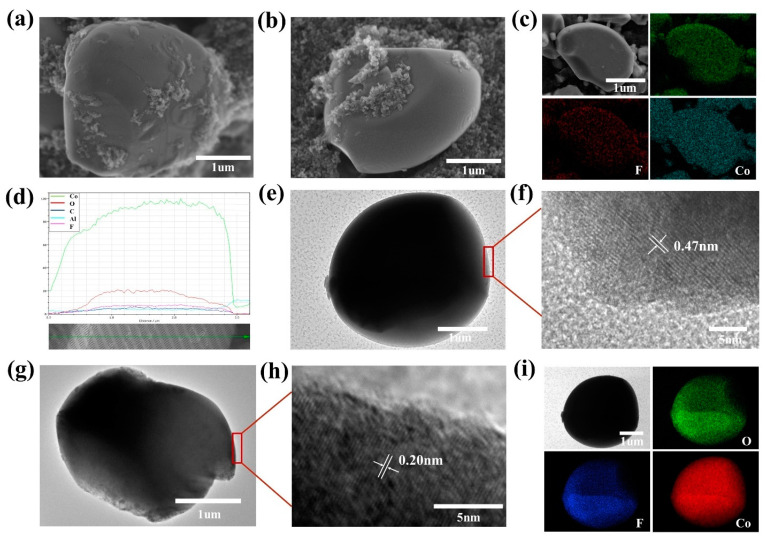
(**a**,**b**) Surface SEM images of pristine LCO and LiF-coated LCO. (**c**) Elemental mappings and (**d**) linear scan plots of LiF-coated LCO. (**e**) Bright filed TEM and (f) HRTEM images of pristine LCO. (**g**) Bright filed TEM, (**h**) HRTEM, and (**i**) elemental mapping images of LiF-coated LCO.

The morphology of the pristine LCO and LiF-coated LCO electrodes is shown in Figure 5a,b. The primary particles of LCO are irregular, but the surface of grains is smooth for uniform coating. Since the thickness of LiF coating is only ~50 nm. No obvious morphology differences can be found between pristine LCO and LiF-coated LCO, implying that the LCO electrode is not affected by the LiF coating. The F element is clearly observed in the elemental mapping (Figure 5c), and the linear scanning (Figure 5d) implies that the F element is evenly distributed on the surface of LCO electrode, confirming the successful coating of LiF on the LCO particles. The morphology of pristine LCO is checked by transmission electron microscopy (TEM) as shown in Figure 5e,f. The obvious lattice fringes of 0.47 nm are indexed as the (003) crystal plane of LCO. The morphology of LiF-coated LCO and HRTEM image are shown in Figure 5g,h. The accordant lattice fringes with the interplanar spacings of 0.20 nm are indexed as the (200) crystal plane of LiF. The F element is evenly distributed on the LCO grain surface as detected by the elemental mappings (Figure 5i), indicating that the LiF coating is homogeneously coated on the surface of LCO particle.

**Figure 6:**
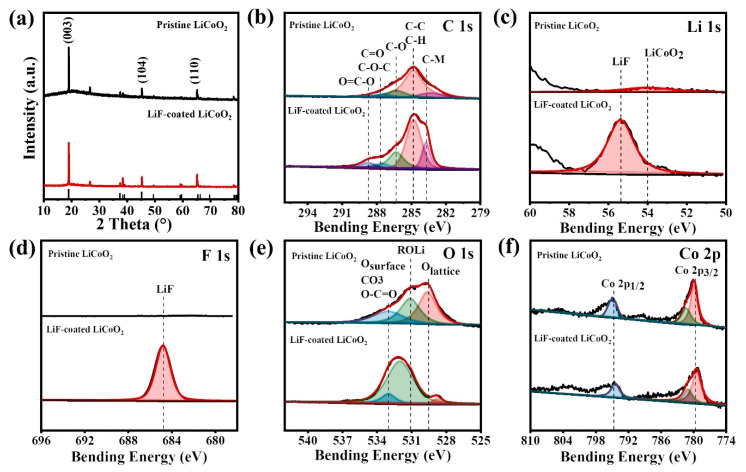
(**a**) the XRD of pristine LCO and LiF-coated LCO. (**b**–**f**) XPS spectra of the C 1s, Li 1s, F 1s, O 1s, and Co 2p photoemission lines collected from LCO and LiF-coated LCO electrodes.

The XRD patterns of LiF-coated LCO and pristine are compared in Figure 6a. Due to the tiny amount of LiF thin film and its poor crystallinity deposited by sputtering, no additional peaks except for LCO can be found in the samples. The LCO powder shows the typical orientations of (003), (104), (006), etc. which is accordant with the reported results. The surface chemical depositions of pristine LCO and LiF-coated LCO electrodes are studied by XPS analysis. The C 1s, Li 1s, F 1s, O 1s, and Co 2p spectra are shown in Figure 6b,f. The C1s peaks (Figure 6b) in the two samples are similar and shifted to 284.8 eV for standardization. It is worth noting that no F signal is found in the pristine LCO due to the replacement of PVDF into CMC binders to avoid the confusion of F from LiF or electrodes. The Li 1s peak (Figure 6c) and F 1s peaks (Figure 6d) of the deposited LiF film are 55.3 and 684.8 eV, respectively. Both results are consistent with the reported bulk LiF materials [29]. The O 1s spectra (Figure 6e) of LiF-coated LCO sample change and move to higher binding energy. The peak of O lattice is weak and the peak of surface O/CO3O-C=O becomes extensive. Figure 6f shows the evolution of the Co 2p peaks of LCO and LiF-coated LCO electrodes. The main peak positions of Co 2P_2/3_ at 777.3 eV and Co 2P_1/2_ at 792.5 eV move to higher binding energy and the peak intensity decreases, which is caused by the formed interfaces between LiF coating and LCO electrodes.

**Figure 7:**
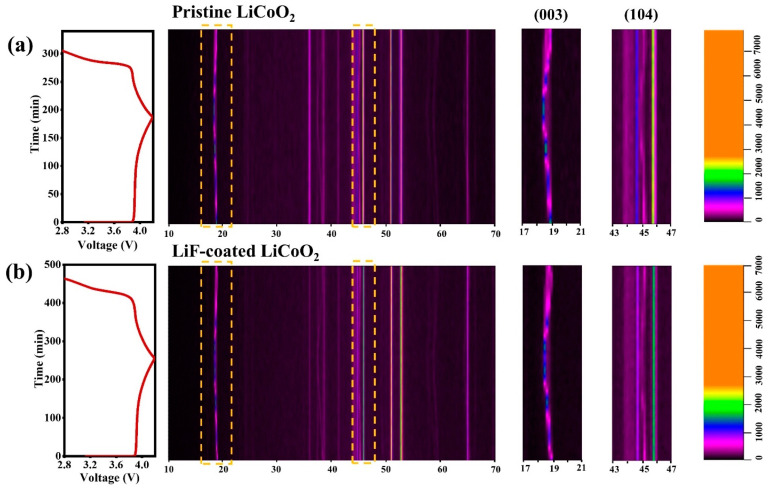
In situ XRD of (**a**) pristine LCO and (**b**) LiF-coated LCO.

In addition, in situ XRD is carried out to study the structural stability and phase transition behaviors of pristine LCO and LiF-coated LCO. The evolutions of diffraction peaks including (003), (104), (006) and (101) facets in the first cycle are shown in Figure 7a,b. In the initial charge, a series of phase transitions occur from H1 to H2, M1, H3 (>4.2 V) and O1 phases, which are closely related to the cut-off voltage and electrochemical signatures [30,31]. The (003) diffraction peak gradually moves to lower angles in the charge process of pristine LCO, and goes back to original states during discharge. The H1 phase transforms to H2 and M1 phases between 3.93 and 4.12 V, indicating that the C-axis was elongated, which was attributed to the repulsive force between the CoO_6_ layers [32]. However, the peak shift of LiF-coated LCO is smaller upon charge implying that the LiF coating slows down the phase transformation. In the initial discharge, the (003) peak returns to the original position without intensive signals. The position of (104) diffraction peak also changes more slightly in the LiF-coated LCO sample compared to pristine LCO. As a result, the peak shift of pristine LCO occurs earlier than that of LiF-coated LCO, confirming the enhanced structural stability after surface coating. Furthermore, the smaller change of peak intensity reflects the smaller volume change of LiF-coated LCO upon the cycling. The results indicate that the surface modification of LiF can effectively inhibit the phase transition and stabilize the structure of LCO electrodes.

## 4. Conclusions

In this work, LiF film is sputtered on the surface of LCO electrodes to improve the electrochemical performance and reduce the voltage polarization. The constituted electrochemical and concentration polarization is theoretically analyzed and proved to be correlated with charger transfer resistance and Li-ion diffusion coefficients. The decreased charger transfer resistance and enhanced Li-ion diffusion kinetic of LiF-coated LCO is indicative of the reduced polarization by the LiF coating. Consequently, the overpotential of LiF-coated LCO is significantly lower than that of pristine LCO as confirmed by various electrochemical tests. As a result, the LiF-coated LCO exhibits higher initial CE of 98.48%, higher capacity retention of 76.9%, and lower overpotential of 0.07 V than those of pristine LCO after 100 cycles. The strategy of coating Li-compounds on electrodes is potential for improving the cycling stability and long-term safety of cathode materials in LIBs.
